# Prevalence of cesarean sections in swiss Bernese Mountain Dogs (2001–2020) and identification of risk factors

**DOI:** 10.1186/s13028-022-00664-9

**Published:** 2022-12-28

**Authors:** Magdalena Schrank, Marco Sozzi, Antonio Mollo

**Affiliations:** 1 Department of Animal Medicine, Production and Health, University of Padua, Viale dell’Università 16, 35020 Legnaro, PD Italy; 2 Department of Land, Environment, Agriculture and Forestry, University of Padua, Viale dell’Università 16, 35020 Legnaro, PD Italy

**Keywords:** Breeding, Canine, Cesarean section, Reproductive parameters, Stillbirth

## Abstract

**Background:**

Dystocia is an important limiting factor in animal breeding due to its cost, stress for the mother and risk of death for the neonates. Assessment of incidence and characteristics of dystocia and the inherent risk of Cesarean section are of major importance. The aim of the present study was to evaluate the reproductive performance of Bernese Mountain Dogs in Switzerland, with a particular focus on the prevalence of Cesarean sections due to dystocia, and identification of possible risk factors.

**Results:**

The investigated population included 401 bitches, 207 sires, and 1127 litters. Litter size was significantly influenced by age and parity of the dam. Incidence of Cesarean section was 30.4%, with 2.0% of procedures being elective. History of previous Cesarean section, age of the dam, and a small litter size significantly influenced the risk for Cesarean section. The stillbirth rate was 12.0%, and the number of stillborn pups was significantly higher for litters delivered by Cesarean sections after birth of the first pup. The inbreeding coefficient had a low to non-significant impact on all reproductive parameters (e.g., litter size, number of stillborn pups).

**Conclusion:**

The sample of Bernese Mountain Dogs of our study had an increased prevalence of Cesarean sections compared to the literature, and advanced age of the dam, litter size and prior Cesarean sections in the dam’s reproductive history was identified as significantly influencing factors. In order to improve pups’ survival rate, elective Cesarean section may be indicated in bitches that have had a previous Cesarean-section/s, are of advanced age, and/or have a small litter.

## Background

The Bernese Mountain Dog (BMD) is one of four Swiss Mountain Dog breeds which has been traced back to more than a century ago in Switzerland [1]. BMD were historically used as farm dogs and their population size has increased continuously over the last few decades due to their increased popularity as companion animals. Reproductive parameters and reproductive performance have been evaluated for selected breeds [2–5] as well as for stray dogs [6]. Evaluation of reproductive parameters for a specific breed or a selected population is valuable as it provides a better understanding of a population, helps to identify problems and decide corrective measures. Dystocia is rather common in dogs [7–9]. The success rate of medical treatment of dystocia is poor and most non-obstructive dystocia cases require Cesarean section (C-section) [10]. Therefore, knowledge about risk factors for dystocia may be of great importance for breeders as well as veterinary practitioners.

Risk factors for canine dystocia include age, litter size, and parity [11–14]. Within specific breeds, the occurrence of physical traits such as brachycephaly has been reported as a risk factor for dystocia [15, 16], thereby contributing to an increase in elective C-sections.

The present study provides an assessment of the reproductive parameters and reproductive performance of a large sample of BMDs in Switzerland over the last 20 years, with the primary aim of investigating the incidence of C-sections, the identification of risk factors for the performing C-section and its outcome. Based on the results, we aim to provide advice in order to increase positive reproductive outcomes such as a decreased number of stillborn pups.

## Methods

### Materials

Data were collected in collaboration with a Swiss breeding club for BMDs (KBS) whose members of the committee reported an increased incidence of C-sections within their population. The KBS provided access to the DogBase database (TG-Verlag Beuing GmbH, Germany), birth announcements (AoB) and mating announcements (AoM). The entries in the DogBase database include general information on bitches and sires such as the date of birth, the inbreeding coefficient (IC), and the results of mandatory health exams, the pedigree as well as information on their offspring. Breeders are required to provide the KBS committee with printed copies of AoB (information on the litter, including dam, sire, date of birth, litter size number of male and female pups and number of stillborns) and AoM (a mating declaration with historical information on the dam, sire, number of matings, and mating dates). Both documents are co-signed by the owners of the bitch and male. The information for these categories is provided separately for male and female pups. Information on the type of parturition (eutocic birth or C-section) is also provided by the breeder of the female indicating the reason for the C-section. Data collection included bitches that were born after January 1st 2000 and their respective litters. No similar inclusion criteria regarding the date of birth of the sire was set. Only litters with availabe AoB (for the litter) and DogBase (for the parents) data were included in the study.

### Statistical analysis

A dataset of collected data was developed using Microsoft^®^ Excel for Mac (Version 16.16.25). Data collected from the previous three sources were cleaned by removing outliers for each detected variable. Data were characterized by exploratory data analysis (regression analysis and distribution analysis). Outliers were identified by a threshold of ± 3 standard deviations. Viable males and females and stillborn pups were reported as percentage of each litter in order to remove the effect of the number of pups in the litter. Gender analysis on single-pup litters was not performed. Statistical analysis was performed with Python 3.6 using Pandas, Statmodels, Pingouin and Bioinfokit packages. Statistical differences between variables were assessed using one-way analysis of variance with covariates (ANCOVA) and Tukey’s post-hoc test with significance level set at 0.05. T-tests with a significance level of 0.05 were performed only if two variables were analyzed. In addition, each variable was tested for normality and homoscedasticity with the Shapiro–Wilk and Levene tests, respectively. Non-normal datasets and variables with different variance were managed by removing outliers (± 3 standard deviations different from the mean) and logarithmic transformation. The results of t-tests were used to identify possible influencing factors focusing on the incidence of C-sections. Numerical results are showed as mean ± 1 standard deviation. Effects of individuals and age were considered as covariates by performing ANCOVA.

## Results

A total of 401 bitches producing 1127 litters between November 2001 and December 2020 were analyzed. The yearly litter distribution is shown in Table 1. The litters were sired by a total of 207 males. Of all the litters included, 784 (69.6%) were born via eutocic parturition, whereas 343 (30.4%) were delivered by C-section. Considering that only 23 of these C-sections (accounting for 6.7% of all C-sections and for 2.0% of all litters) were declared as elective procedures, preplanned and emergency C-sections were not separated for statistical evaluation. The age of dam and sire at the time of whelping ranged between 1.5 and 8.2 years (mean: 3.9 ± 1.5 years) and between 1.4 and 10.1 years (mean: 4.3 ± 1.7 years), respectively. Inbreeding coefficients ranged between 0 and 7.8 (mean: 1.3 ± 1.3) for dams, and between 0 and 8.8 (mean: 0.8 ± 1.2) for sires. Bitches gave birth to 1–8 litters (mean: 2.8 ± 1.5 litters) within the study period, while males sired 1–38 litters (mean: 5.5 ± 6.5 litters). A total of 8106 pups were born with 3996 (49.3%) being recorded as males and 4110 (50.7%) as females and a mean of 3.5 ± 2.0 and 3.6 ± 2.0 male and female pups per litter, respectively. A total of 6098 pups were born through eutocic parturition, whereas the birth of the remaining 2008 pups required a C-section. Litter size varied greatly between 1 and 16 pups with a mean of 7.2 ± 3.1 pups. Overall, 973 pups were stillborn, resulting in a mean of 0.9 ± 1.1 stillborn pups per litter and an overall rate of stillbirth of 12.0% for all the bitches included. A total of 397 litters (35.2%) were born from primiparous bitches, while 730 (64.8%) were born from pluriparous bitches. Similar results were observed for C-sections, with a distribution of 34.4% and 65.6% born from primiparous and pluriparous bitches, respectively. Litter size differed between primiparous and pluriparous bitches, with primiparous bitches giving birth to larger litters (7.6 ± 3.0 pups per litter) than pluriparous bitches (6.9 ± 3.0 pups per litter) (F = 10.1; P < 0.001). Dam age influenced litter size, with younger bitches giving birth to significantly larger litters than older bitches (F = 29.4; P < 0.001). In addition, bitches with one or more previous C-sections had significantly smaller litters (5.8 ± 3.0 pups) than those without a previous C-Sect. (7.5 ± 3.0 pups) (F = 34.9; P < 0.001). Sire’s age at the time of parturition did not significantly influence litter size. When considering data on stillborn pups, we observed that the inbreeding coefficient had no significant impact at the dam (F = 0.8; P > 0.05), sire (F = 1.2; P > 0.05), or litter level (F = 1.3; P > 0.05). The number of stillborn pups increased with increasing litter size (F = 2.1; P = 0.008) and was significantly different when evaluating parity of the dam (F = 4.4; P = 0.04).

**Table 1:** Yearly distribution of number of litters, Cesarean-sections and the annual incidence of Cesarean-section

Year	Number litters	Number c-sections	%
2001	2	0	0.0
2002	16	6	37.5
2003	34	7	20.6
2004	52	13	25.0
2005	60	15	25.0
2006	69	12	17.4
2007	75	22	29.3
2008	72	19	26.4
2009	89	25	28.1
2010	64	13	20.3
2011	66	17	25.8
2012	69	15	21.7
2013	63	20	31.7
2014	59	14	23.7
2015	56	15	26.8
2016	60	28	46.7
2017	62	29	46.8
2018	49	23	46.9
2019	59	24	40.7
2020	51	26	51.0
Total	1127	343	30.4

Data on mating were collected from 604 available AoMs that had announced mating between November 2012 and September 2020, of which 440 (72.9%) resulted in successful whelpings, while 164 (27.1%) were unsuccessful. The mean number of matings differed only slightly between successful (2.3 ± 0.8) and unsuccessful (2.1 ± 0.8) breeding attempts. The number of matings within the same oestrus ranged between 1 and 5, regardless the outcome. In most cases, bitches were bred twice with a mean interval between matings of 1.5 ± 0.6 days. A comparison of the interval between the first and the last breeding within each cycle with positive and negative outcomes is reported in Table 2. The number of repeated matings of the same male with the same female in subsequent heats was evaluated. This parameter was considered either as a repetition of mating (RoM) when following an unsuccessful breeding attempt or as a repetition of litter (RoL) when following a successful breeding attempt. Overall, repetitions made up 16.1% of all the AoMs in the dataset; 48.5% of these were RoL, whereas 51.5% were RoM. Repetitions of litters were generally performed only once, although two bitches had two RoLs each. RoM was performed once in 76.7% of cases (range 1–3 times). In both RoL and RoM, the majority of bitches became pregnant. The gestational length calculated from the first date of mating ranged from 57 to 70 days (mean: 62.7 ± 1.9 days). When calculating from the last date of mating, the gestational length ranged from 55 to 69 days (mean: 60.7 ± 1.9 days). Overall, 58.6% of all the litters were born 63 ± 1 days after the first mating.

**Table 2:** Overview of the interval between first and last mating in successful and unsuccessful breeding attempts

n° of matings	Pregnant		Not pregnant	
	n° dams	Interval (media ± SD)	n°dams	Interval (media ± SD)
1	60		42	
2	229	1.5 ± 0.6 days	71	1.4 ± 0.6 days
3	131	2.8 ± 0.9 days	48	2.8 ± 0.9 days
4	18	3.8 ± 1.1 days	1	4.0 ± 0.0 days
5	2	5.0 ± 0.0 days	2	4.5 ± 0.7 days

n° of matings = number of matings during one oestrus cycle; n° dams = number of dams; Interval (media ± SD) = Interval between the first and the last mating given as a media ± SD in days

Of the 401 bitches included in the analysis, 218 had at least one C-section over the studied period or their reproductive career. Up to a maximum of four C-sections in certain subjects (Table 3) were recorded. When considering the occurrence of C-section, 57.3% of bitches had only one, 30.3% had two, 10.1% had three, and 2.3% (five bitches) had four C-sections. Of the 183 bitches (45.6%) that did not have any C-sections, 50.8% had either one or two litters. Of all males, 62.3% sired at least one litter that was born by C-section. Two males had a particularly high incidence of C-sections in their sired litters, with 43.8% of 32 sired litters for one male and 52% of 25 sired litters for another male. Information on the timing of C-section was available for 41.1% (n = 1. 41) of all C-sections, with 41.8% (n = 59) performed prior to the birth of the first pup (T0) and 58.2% (n = 82) performed after the birth of the first pup (T1). The evaluation of previous C-sections showed that 58.9% of the 209 litters born from bitches which had at least one C-section previously in their reproductive history, were again delivered via C-section. A significant increase of the number of C-sections in bitches that had at least one prior C-section (F = 80.6; P < 0.001) was observed. Bitches which had previous C-section/s in their history had also a significantly higher IC (1.5 ± 1.3) compared to bitches which did not (1.2 ± 1.2) (P = 0.009). Furthermore, bitches undergoing C-sections were significantly older (P < 0.014). Litters born via C-sections were significantly smaller than litters born via eutocic parturition, with mean litter sizes of 5.9 ± 3.3 and 7.8 ± 2.8 pups, respectively (P < 0.001). Other factors (age of the sire, parity of the dam, inbreeding coefficient of the bitch, sire, and litter) were not associated with dystocia. Interestingly, the number of stillborn pups did not significantly differ between eutocic parturitions and C-sections (P > 0.05). Although the presence of previous C-sections had a significant influence on litter size, no such influence of prior C-sections was observed on number of stillborn pups.

**Table 3:** Overview and distribution of number of Cesarean-sections related to the number of litters bitches had

Number of litters	Number of Cesarean-sections											
	0 CS		1 CS		2 CS		3 CS		4 CS		total	
	n°	**%**	n°	**%**	n°	**%**	n°	**%**	n°	**%**	n°	**%**
1	47	**53.4**	41	**46.6**							88	**100**
2	46	**43.8**	32	**30.5**	27	**25.7**					105	**100**
3	35	**41.2**	23	**27.1**	18	**21.2**	9	**10.6**			85	**100**
4	29	**43.9**	19	**28.8**	10	**15.2**	6	**9.1**	2	**3.0**	66	**100**
5	17	**44.7**	6	**15.8**	8	**21.1**	5	**13.2**	2	**5.3**	38	**100**
6	9	**64.3**	2	**14.3**	2	**14.3**	1	**7.1**			14	**100**
7			2	**50**			1	**25**	1	**25.0**	4	**100**
8					1	**100**					1	**100**
Total	183	**45.6**	125	**31.2**	66	**16.5**	22	**5.5**	5	**1.2**	401	**100**

n°=number; percentages evidenced in bold

The evaluation of timing of C-section revealed a highly significant difference in number of stillborn pups (P < 0.001). In C-sections performed at T0, a mean of 0.3 ± 0.7 stillborn pups was reported compared to a mean of 1.2 ± 1.2 stillborn pups in C-sections that were performed at T1. Although parity had no impact on timing of C-section, the age of the bitch did. Bitches undergoing C-section at T1 were significantly older than those undergoing surgery at T0 (P = 0.014). Furthermore, significant differences were observed regarding the presence or absence of previous C-section/s in the dams reproductive history (P = 0.029) as well as in her litter size (P < 0.001). Bitches without previous C-sections were more frequently taken to surgery at T1. Differences in litter size were not only highly significant, but also very evident, with a mean litter size of 3.1 ± 2.7 pups in C-sections done at T0 vs. a mean litter size of 7.0 ± 2.8 pups in C-sections done at T1.

## Discussion

The breeding data used in the present study were not provided by a veterinary professional, yet their source is considered trustworthy as it is objective information obtained from an official body and which are not directly connected to a specific veterinary exam or dexterity. Furthermore, the obligation given by the KBS to provide both AoM and AoB [17] ensures that collected data include all litters born in a certain period of time. Litters of BMDs born in Switzerland without being tracked by AoM, AoB or DogBase entries are considered to be the product of non-FCI (Federation Cynologique Internationale) breeding, are not subject to the same rules as the litters included in the dataset and are, therefore, not (or only to a limited extent) comparable to the litters that were obtained following a certain generally accepted method [17]. Such uniformity allowed to develop a large dataset of a single breed, with parameters which are easily comparable. Although these regulations provided an advantage, they need to be considered in the interpretation of the results and when comparing them to the literature, in particular regarding the age of the dam, the number of litters per bitch, and the IC of the litter. The KBS imposes a rule that bitches may only reproduce until 8 years of age [17] with only few exceptions whereas no age limit is imposed on sires. Males may sire six litters per year, while bitches may give birth to one litter per year [17]. The inbreeding coefficient of the litter is calculated based on five generations and may not exceed 6.25% [17].

Mating data were collected exclusively using the AoM, which does not provide information on ovulation staging. No information was requested or provided about whether the bitch mated naturally or via artificial insemination, so no conclusions may be drawn in this regard. The use of artificial insemination is regulated by the FCI and is only permitted in bitches which have reproduced previously by natural mating [18] which means that the first litter of each bitch was evidently conceived without artificial insemination. The mating of the same male to the same female may be repeated once in cases of a satisfactory outcome (RoL) or up to 3 times in case of failure of the bitch to become pregnant (RoM)[17]. RoL and RoM were roughly equivalent, 49.5 and 51.5% of all breedings, respectively. Frequently, an agreement is made for a RoM without repeated payment to the owner of the sire. This would explain the high number of RoMs and the fact that more than 75% of all the RoMs were repeated only once. Failure to conceive in the bitch is frequently due to breeding management, abnormal cyclicity and also to pathologies or abnormalities within the reproductive tract. Although it is impossible to extrapolate what caused failure to conceive in 51.5% of mated bitches, a > 50% frequency of failure to conceive in these dogs is a reason for concern and it should be investigated. This is the first report of the frequency of RoL and RoM in a canine breed.

The interval between the first and the second mating was 1.5 ± 0.6 days in all bitches (regardless of whether or not becoming pregnant) and, as such, lower than the frequently advised interval of 48 h. This shorter interval compared to what should normally be done is probably a consequence of limitations in organization and availability of help, as most breeders included in the dataset do not breed dogs as their main sources of income and may, therefore, have difficulties staying close to the selected male for more than 2 days. Gestation length was calculated based on the interval between mating and parturition. The majority of bitches gave birth 63 ± 1 days after the first mating. This is in accordance with the gestational length reported after ovulation [19]. Assuming that in the bitches of our study (a) ovulation staging was not done on a regular basis (which may justify the high rate of failure to conceive) and (b) general and reproductive health were normal, the high frequency of gestations of 63 ± 1 days indicates that (i) most breeders mated their bitches at the time of ovulation and (ii) an equally high percentage of bitches does not ovulate when expected. The possibility to obtain data on ovulation staging would be of great importance and further data collection is necessary considering that there might be a correlation between gestational length and the risk of C-section.

Litter size was 7.2 ± 3.1 pups and differed depending on parity and age of the dam, with younger bitches and primiparous bitches giving birth to larger litters than older and pluriparous dams. The mean litter size of this study was similar to [20–22] or higher than [23–26] what reported for BMD in the literature. Female fertility is higher at a younger age [5, 20, 23]. A possible influence of parity on litter size was previously reported [5, 23], although results differ between studies and/or breeds. Borge et al. [23], for example, found that primiparous bitches produced significantly larger litters. An increase in litter size between the first and second litter was observed in the Drever breed [5], followed by a continuous decrease from the third litter onwards. This pattern is similar to the BMD, characterized by an increasing litter size up to the third litter followed by a decrease in subsequent litters. In the present study litter size was compared only between primiparous and pluriparous bitches without considering parity. A direct comparison with the data described in the literature is therefore not possible. It has to be considered that the majority of the bitches within the present study were bred relatively early in life (mean age at birth: 3.9 ± 1.5 years), which suggests that prolificacy of bitches within the present dataset should be interpreted taking into consideration both age and parity of the dam.

The IC influences reproductive parameters such as litter size, stillbirth and neonatal mortality [20, 25]. Significantly larger litters are produced from bitches with IC of < 6.25% compared to bitches with ICs of 6.25–12.5% and over 12.5% [25]. Although dams with inbreeding coefficients over 6.25% were present in this study, the maximum was 7.8% and the mean was 1.3%. As the KBS in general does not allow breeding of dogs with an IC higher than 6.25% no significant differences in IC at the dam, sire, or litter levels were observed in the present study. The only significant difference in the evaluation of inbreeding coefficients was observed when comparing bitches that had previous C-sections with bitches that did not. The relationship between IC and the risk of having a C-section is probably multifactorial therefore further investigations are needed on this respect. The overall sex distribution within litters (49.3% males *versus* 50.7% females) was similar to what has previously been reported in the literature [5, 27]. The incidence of stillbirth was 12%, which is higher than what has been previously reported for various breeds [20, 28, 29] and was significantly different between pluriparous and primiparous bitches of our study. A higher number of stillborn pups in primiparous bitches may be attributed to longer parturitions and less experience of the bitch, yet also the experience of the breeder which is assisting has to be taken into consideration. Further data collection is necessary to confirm such hypothesis.

The evaluation of the risk of C-sections within the dataset was the main aim of this study. Overall, incidences of 30.4% of C-sections and a mere 2% of elective C-sections were observed. When questioned about the low number of elective C-sections the KBS committee hypothesized that despite the perceived high risk of a C-section in this breed, most breeders try to avoid surgery thus letting the bitch proceed with a trial of labor and intervening only when a problem arises. The rate of C-sections within the dataset was higher than what was previously reported for the BMD [30] as well as for other breeds [29, 31]. In addition, more than half of all the bitches had at least one C-section in their reproductive career. Similar results have been observed the sires of our study, with some sires having an incidence of C-sections within their sired litters of up to 50%. Prior C-sections within a bitch’s reproductive history had a significant influence on the incidence of C-sections in subsequent parturitions - a finding that is in agreement with what has previously been described [12, 32]. Furthermore, our results indicate that bitches with a previous C-sections have smaller litters. Smaller litters and bitches of advanced age were found more frequently in the group of C-sections compared to the group of litters born via eutocic parturitions. Therefore, a possible additive effect of these factors in increasing the risk of C-sections cannot be excluded. A higher risk of C-section for litters of one or two pups has been reported [29], possibly due to an increased fetal size - which may result in a disproportion of the fetus [33, 34] or the birth canal - or due to insufficiency of fetal signaling for the induction of labor [29]. The presence of a singleton or two pups should be a reason to advise the owner to request an elective C-Sect. [11]. Interestingly, a statistically significant difference in stillbirths between C-sections and eutocic parturitions was not observed, yet the timing of C-sections is important as confirmed by the observation that significantly more pups were stillborn in C-sections performed at T1 than in C-sections at T0. Such results have been previously reported [7, 24, 29] and might be explained by prolonged fetal stress with prolonged stage 2 parturition. Litter size differed significantly as well, being significantly smaller with C-sections performed at T0 which confirms the observation that litters of one or two pups are at higher risk of dystocia than larger litters. Although the BMD is not generally considered a breed at risk for dystocia, further research is needed to determine whether differences in the phenotypical appearance following changes in the selection processes may have contributed to an increase in dystocia and consequent emergency C-sections within the studied population. Breeding of BMD in Switzerland is highly regulated as described above, yet no rules are imposed regarding the maximum number of C-sections a bitch may have prior to exclusion from breeding. Such a lack of regulation certainly rises an ethical concern, as does high incidence of emergency C-sections in bitches of breeds which are unlikely of or unable to whelp naturally. Regulations in this regard are not imposed by the FCI but may be imposed by the national breeding clubs. Limitations of the number of C-section a bitch may have would be useful, yet may not be considered as a solution. Investigation of the underlying conditions or reasons for an increased incidence of C-section or the inability to whelp naturally is of great importance in order to implement successful breeding strategies.

## Conclusion

The collection and elaboration of data on the BMD in Switzerland proved that this population has an important incidence of C-sections, with increased incidences in bitches that had prior C-sections. Furthermore, the timing of C-sections in this population had a significant impact on the number of stillborn pups. The BMD is not generally considered a breed at-risk of C-section, yet elective C-sections may be considered in pregnancies (a) of bitches of advanced age, (b) of bitches which have previous C-section/s in their reproductive history and /or in (c) pregnancies with a low number of pups thereby improving puppy survival. Furthermore, these results are reason to advise the meticulous evaluation of subsequent breeding attempts in bitches with a history of prior C-sections.

## Data Availability

The datasets used and/or analyzed during the current study are available from the corresponding author on reasonable request.
